# Neural correlates of gratitude

**DOI:** 10.3389/fpsyg.2015.01491

**Published:** 2015-09-30

**Authors:** Glenn R. Fox, Jonas Kaplan, Hanna Damasio, Antonio Damasio

**Affiliations:** Department of Psychology, Brain and Creativity Institute, University of Southern CaliforniaLos Angeles, CA, USA

**Keywords:** affective neuroscience, fMRI, Holocaust testimony, pro-social behavior, altruism

## Abstract

Gratitude is an important aspect of human sociality, and is valued by religions and moral philosophies. It has been established that gratitude leads to benefits for both mental health and interpersonal relationships. It is thus important to elucidate the neurobiological correlates of gratitude, which are only now beginning to be investigated. To this end, we conducted an experiment during which we induced gratitude in participants while they underwent functional magnetic resonance imaging. We hypothesized that gratitude ratings would correlate with activity in brain regions associated with moral cognition, value judgment and theory of mind. The stimuli used to elicit gratitude were drawn from stories of survivors of the Holocaust, as many survivors report being sheltered by strangers or receiving lifesaving food and clothing, and having strong feelings of gratitude for such gifts. The participants were asked to place themselves in the context of the Holocaust and imagine what their own experience would feel like if they received such gifts. For each gift, they rated how grateful they felt. The results revealed that ratings of gratitude correlated with brain activity in the anterior cingulate cortex and medial prefrontal cortex, in support of our hypotheses. The results provide a window into the brain circuitry for moral cognition and positive emotion that accompanies the experience of benefitting from the goodwill of others.

## 1. Introduction

How would you feel if in the middle of your most distraught moment, unbound from your every day comforts and scared for your survival, a complete stranger saved your life? When we are the beneficiaries of good human conduct, we can experience feelings of gratitude. The importance of gratitude and its benefit to sociality is stressed in philosophy and in religion. Cicero cited gratitude as the mother of all virtues, and the Roman Stoic Seneca conceived of gratitude as a fundamental motivational drive, critical for building interpersonal relationships. As a research theme, however, empirical investigations of gratitude are relatively rare (Emmons and McCullough, 2004), although this is beginning to change (Watkins, 2014). It has been shown that gratitude can be generated by gifts that largely fulfill two criteria: (1) they come as a result of perceived genuine effort from the giver and (2) they are valuable and fulfill important needs for the recipient (Tesser et al., 1968). Recent studies have shown that gratitude is associated with benefits to subjective well-being (Emmons and McCullough, 2003; Froh et al., 2008), increased resilience to trauma (Kashdan et al., 2006) and benefits to social relationships (Algoe et al., 2008; Lambert et al., 2010). Individuals vary in how grateful they tend to be, and those who are more grateful show enhanced psychological well-being (Wood et al., 2008a, 2009). The results from psychological investigations of gratitude have laid a foundation for what can be expected when we facilitate the experience of gratitude.

On the other hand, the cognitive and neural mechanisms behind the experience of gratitude itself have rarely been studied (Wood et al., 2008b). An investigation of the neural basis of gratitude extends the reach of affective neuroscience beyond the study of basic emotions into the complex social emotions that are important for well-being. At the level of the brain, the investigation of the generation and experience of gratitude is just beginning. One study found that making moral judgments involving gratitude elicited activity in the right anterior superior temporal cortex (Zahn et al., 2008). One study of brain morphology found that individual differences in proneness to gratitude correlated with increased gray matter volume in the right inferior temporal gyrus and posteromedial cortices (Zahn et al., 2014). Another recent study found a correlation between individual differences in a genotype for oxytocin function and behavioral expressions of gratitude (Algoe and Way, 2014), pointing to gratitude's importance in social bonding. In a study of admiration and compassion, participants reported being grateful for their own well-being when they processed stories that evoked compassion for emotional pain, which is associated with brain activity in cortical midline structures such as the posteromedial cortices (Immordino-Yang et al., 2009). It is unknown, however, how the brain generates the range of feelings associated with gratitude. Knowledge of what the brain is doing during the experience of gratitude provides a window into gratitudes relationship to mental health and resilience (Wood et al., 2008b; Huffman et al., 2014). Examining the neural correlates of gratitude is relevant to the design of interventions for practicing gratitude and can resolve questions regarding the respective roles of reward and moral cognition in gratitude (Emmons and McNamara, 2006).

Gratitude is a social emotion that signals our recognition of the things others have done for us (Emmons and McNamara, 2006). The expression of gratitude may serve to communicate reciprocal engagement and to prevent being seen as a “free-loader,” which could end in social punishment (de Quervain et al., 2004). Gratitude then, is an emotion that not only enhances our social relationships (Algoe et al., 2008), but also signals to others a recognition that we are a fair partner (Sigmund, 2007). It is an emotion critical to maintaining social standing, to indicate when we have received benefit, to reinforce beneficial behavior toward the recipient, and to motivate prosocial behavior in the future (McCullough et al., 2008).

The systematic identification of the thoughts, feelings and behaviors associated with gratitude is a difficult endeavor given the dramatically different reactions people have, even when experiencing similar exchanges. In addition, the scale of gratitude is wide; it can be as small as the gratitude felt for someone holding a door for you (Okamoto and Robinson, 1997), or it can be overwhelming as in the case of life-saving gifts such as organ donations (Gill and Lowes, 2008). Gratitude can be narrowly focused toward a specific benefactor (Tesser et al., 1968), or can be broad, focused on spirituality and thankfulness for life in general (McCullough et al., 2002; Baetz and Toews, 2009). In the present investigation, we focus on gratitude in the context of gift-giving, involving a donor, a recipient, and a gift; and we focus on the recipient of the gift. We use the term “gift” broadly to refer to both material gifts, such as food or clothing, and non-material gifts in the form of help or psychological support.

The gift-based stimuli used in our experiment were drawn from stories of survivors of the Holocaust, housed in the USC Shoah Foundation Institutes Visual History Archive. The archive is comprised of over 50,000 videotaped testimonies from survivors of the Holocaust. Many survivors tell stories from the midst of this tragedy in which their lives were saved or helped by others through the provision of food, shelter, or clothing. In these stories, the survivors often report strong feelings of gratitude. We selected a collection of these stories and transcribed them into first-person vignettes or scenarios. In the experiment, participants immersed themselves in the context of the Holocaust and experienced these scenarios. We created documentaries detailing the events of the Holocaust aimed at giving the participants an understanding of the Holocaust. Once participants were immersed in the time period, they viewed the series of gifts that were designed to elicit varying degrees of gratitude, and they were asked to imagine how they would feel if they were in the same situation. For each gift, participants rated how much gratitude they felt. Their ratings of gratitude were correlated to brain activity collected using functional magnetic resonance imaging (fMRI).

The reasons for adopting this approach are as follows. In previous studies of the determinants of gratitude, participants have read texts describing scenarios and placed themselves in specific settings while receiving gifts (Tesser et al., 1968; Lane and Anderson, 1976; Wood et al., 2010). We used a comparable text-based approach so that we could eventually compare our results to those in the existing literature. In addition, we used stimuli related to the Holocaust in an attempt to create an experience that would firmly engage the participants in the experiment and thus avoid habituation to the stimuli. The use of narrative-based stimuli to elicit realistic emotional responses in the scanner has also proven effective in prior research on related social phenomena (Immordino-Yang et al., 2009, 2014; Fox et al., 2013).

Our predictions are built around findings from previous psychological research on gratitude in combination with brain imaging studies of related phenomena. We hypothesized that ratings of gratitude would correlate with brain activity in circuits associated (1) with moral cognition; (2) with reward from the pleasure of receiving a benefit in social interactions; and (3) with social cognitive processes such as perspective-taking and theory of mind. Specifically, we hypothesized that the experience of gratitude would relate to changes in activity in the posteromedial and insular cortices, medial prefrontal cortices and nucleus accumbens (Bechara et al., 2000; Knutson and Cooper, 2005; Harbaugh et al., 2007; Immordino-Yang et al., 2009; Van Overwalle, 2011).

## 2. Materials and methods

### 2.1. Participants

Twenty-six participants (13 female; average age: 21 2.21 years, range 18–28) were recruited using USCs psychology subject pool as well as posted fliers and advertisements on USCs University Park Campus. Three participants were removed due to computer and scanner malfunctions, leaving a final sample of 23 participants (12 female). All research participants gave informed consent and all activities were done in accordance with and with approval from USCs Institutional Review Board policies on human subjects research. Participants were right-handed, native English speakers. The participants filled out an open-ended questionnaire regarding their personal experience with the Holocaust. No participants in this sample reported having extensive contact with anyone who went through the Holocaust, or significant educational experience with the Holocaust greater than a single lecture or exposure to the historical events beyond movies or books.

### 2.2. Procedure

The experiment was designed to immerse the participants in the events of the Holocaust, helping them respond to written gift-related stimuli (detailed below) using their own reactions. The experiment took place in four parts; each part dedicated to a different phase of the Holocaust. This approach was designed to mimic the experience of the United States Holocaust Museum, where visitors are asked to imagine living through the events of the Holocaust in the order that they occurred, often categorized into four chronological phases. The four phases were: 1. The rise of Nazism and Persecution, 2. Internment, 3. The Final Solution, 4. Final Months and Liberation. To enhance the context of the stories, the stimuli were designed to be specific to each phase. For example, stories of being helped by the Red Cross during liberation took place in the fourth phase. We chose to present the four phases in chronological order to provide historical context to the participants, to enhance the ecological validity of the experiment, and to maintain the participant's engagement.

Inside the scanner, each phase began with a short, in-house created documentary detailing the events of that phase of the Holocaust. The documentaries were about 2 min long and were created in collaboration with students from the USC School of Cinematic Arts. The documentaries relied on powerful images as well as a professional actor providing a voice-over description. We did not collect fMRI data during the viewing of the documentaries. After each documentary, participants viewed the stimuli related to that phase while we collected fMRI data.

**Figure 1:**
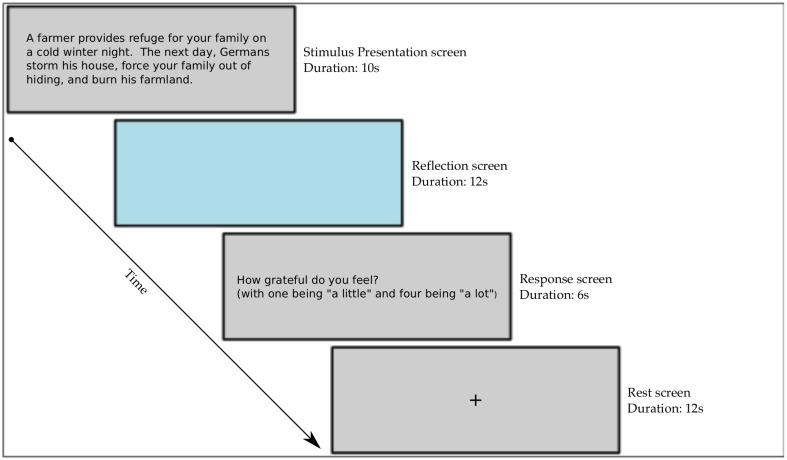
**Scanner stimuli presentation paradigm**.

The task (see Figure 1) consisted of four conditions presented in the following order: stimulus, reflection, probe and rest. Participants read the text of the stimuli on a screen reflected on a mirror mounted on the magnetic head coil. For each stimulus, they were given 10 s to read the text and understand the context of the stimulus. After the stimulus, participants were shown a light blue reflection screen. Participants were told during the reflection screen to feel, as much as possible, how they would feel if they were in the same situation as described by the stimulus. During this time, they were told to imagine themselves in the situation presented and to form as deep, personal and realistic of a reaction as they could. The reflection period lasted 12 s. Following the reflection period, participants rated how much gratitude they felt in response to the event on a scale from 1 to 4. Participants were told to scale their gratitude such that a 1-rating would be associated with a small amount of gratitude, as in receiving lunch from a friend, and a 4-rating indicated events that overwhelmed them with gratitude. Participants were given the option to advance from the stimulus to the reflection period manually, although this occurred on fewer than 1% of the trials. After the rating screen, the participants were given a jittered time of 12–16 s of rest, indicated by a black fixation cross on a light gray screen. This served as the baseline condition for our analyses. During the rest period, participants were told to put everything out of their mind from the previous event and to rest and return to their baseline. They were told to treat each stimulus as an independent event and not to compare their ratings from one event to the others. This was a within-subject experiment, stimuli within each phase were presented in random order for each participant.

After the scanning session, participants were asked to review the stimuli outside the scanner, this time rating each gift according to how much they felt the gift was needed, how much effort they felt the donor had taken to produce the gift and again how much overall gratitude they felt for the gift. The stimuli were designed to elicit varying degrees of gratitude as a product of how much the gift was needed and how much effort it took to provide (Tesser et al., 1968; Lane and Anderson, 1976). Because gratitude is built on these factors, it is possible that need and effort could also explain variance in the brain activity. Participants were told that need was an umbrella term that included the subjective value of the gift, the utility of the gift and also the gift's ability to fulfill important basic and psychological needs. Ratings for effort included the intention of the gift, the cost of the gift and the degree to which the donor's life was affected by giving the gift. We collected the ratings of need and effort to examine their correlation with gratitude, in order to establish a link to previous studies of the factors involved in the generation of gratitude (Tesser et al., 1968; Lane and Anderson, 1976; Wood et al., 2008b, 2011). This analysis was conducted using SPSS version 18. The ratings of need and effort were done post-scan so that the responses to the stimuli during the scan could be focused on gratitude alone.

**Figure 2:**
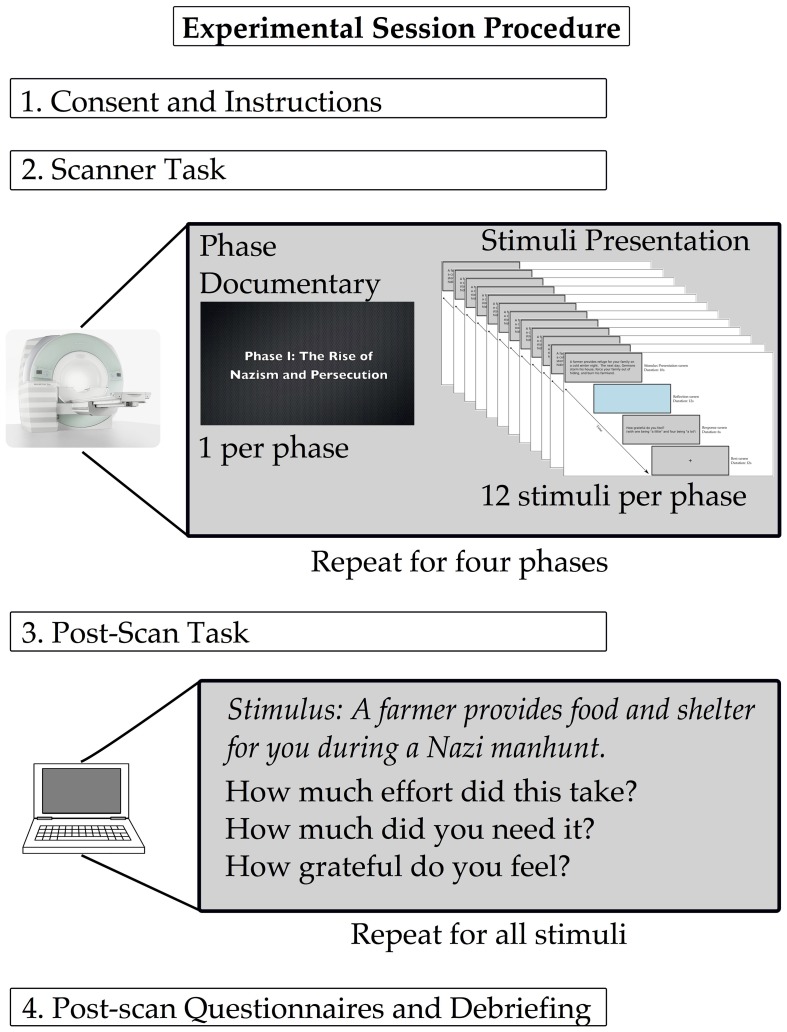
**Experimental session protocol**. The stimuli presentation paradigm for the scanner (shown here in miniature) is detailed in Figure 1.

Participants were asked to fill out personality questionnaires to assess how individual differences in personality affect how a gift was perceived. Participants filled out the Interpersonal Reactivity Index (IRI;Davis, 1983), the six-item gratitude questionnaire (GQ-6; McCullough et al., 2002), the Maslow need scale (Lester, 1990) and the Big Five Personality Index (BFI; John et al., 1991). Participants also completed a homemade questionnaire to assess their experience in the study. They were asked to rate items on a 7-point Likert scale where 1 referred to not at all and 7 referred to completely. The questions were: (1) How involved did you feel in the task/situations, (2) How similar do you think your feelings during the situations match what you would have felt if the experience was real? (3) How difficult was it to put yourself in the situations? and (4) How much do you feel that you have an increased understanding and sense of empathy for the Holocaust from going through this experiment? Following these four questions, we asked the following open-ended questions: (1) Were there any situations or stimuli that you found to be confusing that you can remember? (2) Were there any situations or stimuli that you found to be particularly moving or powerful? (3) What do you think this study was about? Where you focused on figuring this out during the study? (4) Do you have any personal experience or connections to the Holocaust? and (5) Did you have any previous knowledge of the Shoah Foundation Institute? The aim of these questions was to screen for participants whose personal history may have affected their responses and to assess the participant's involvement in the study. The answers to the likert scale questions were analyzed using a one-sample Student's *t*-test to test the hypothesis that the participants rated each question in a way that indicated that they were engaged in the experiment. See Figure 2 for illustration of the order of events in the experimental session.

### 2.3. Stimuli

The goal of the study was to examine a wide range of gratitude experiences in the context of gift-giving. The stimuli consisted of a collection of stories based on testimony from survivors of the Holocaust. The stories were selected from testimony housed in the USC Shoah Foundation Instituteõs Visual History Archive, comprised of 50,000 videotaped Holocaust survivor testimonial. To create the stimuli, research assistants viewed testimonies and selected stories or scenarios in which the survivors tell of moments when aid was given, including shelter, food, clothing, or emotional support.

**Figure 3:**
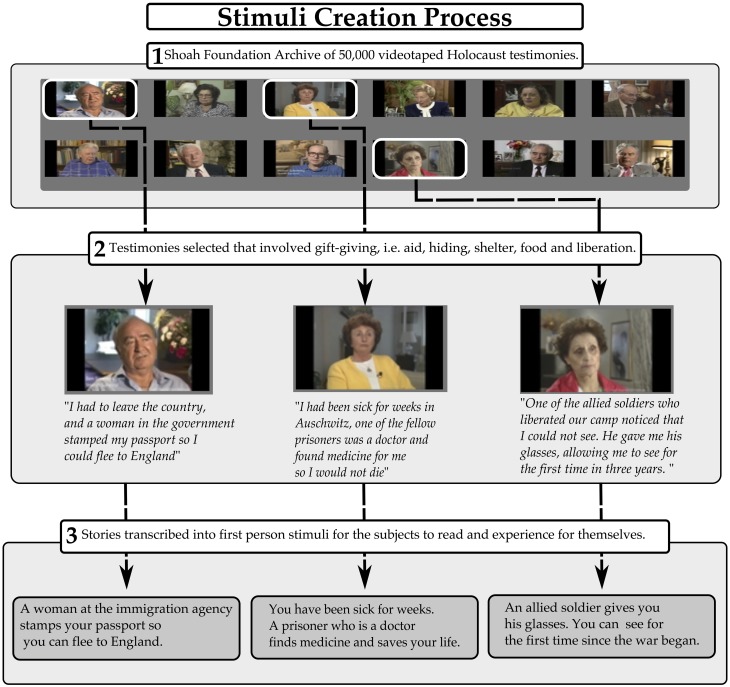
**Stimuli creation process**.

The scenarios described by the survivors were transcribed and condensed into texts ranging from 30 to 40 words and were rephrased to be in the first person. These short texts were used as stimuli. The stimuli were selected to vary according to how much need and effort were involved in the gift. Some gifts were given that fulfilled a high amount of need, but were given with very little effort. For example, during the early phase of the war, a local bakery leaves its unsold and old bread outside in the alley for you to eat. Other gifts came at a high degree of effort, but did not fulfill an important need. An example of this would be a gift in which a bed is offered to you in a concentration camp, but the bed is infested with rodents and insects. One can imagine having some gratitude for each of these gifts, but the reaction for these two gifts is unlikely to be the same. Finally, many of the gifts were given with high need and high effort, such as a fellow prisoner risking her life to steal food from the SS quarters and bring it to you while your are sick in the bunks. Comparing these diverse scenarios allows the investigation to move closer to the actual neural correlates of gratitude, as the range of experiences mimics the real life range of grateful experience. The goal of including these complexities in the stimuli is to leave only the portion of brain activity correlated with the varying experience of gratitude common throughout the stimuli. Through manipulating need and effort independently, we aimed to control for the amount of perspective-taking required, so as to average out confounds related to the success of taking someoneõs perspective and to de-correlate gratitude from simple needs to understand other peoplesõ perspective. The individual responses to each of the stimuli were expected to vary considerably, thus the participant's own responses were used in the analyses. There were a total of 48 stimuli, 12 from each of the four phases of the Holocaust (see Figure 3).

To validate the approach, the stimuli were tested with 42 participants (21 female) in a separate behavioral experiment. In this testing, the participants worked with a booklet of the stimuli and rated each gift according to how much gratitude they felt after receiving the gift, as well as how much they needed the gift and how much effort it took to provide the gift. The testing verified that the stimuli effectively and reliably elicited varied feelings of gratitude and that the stimuli were clear and understandable.

### 2.4. Image acquisition

Functional and structural fMRI were performed at the Dana and David Dornsife Cognitive Neuroscience Imaging Center at USC on a Siemens 3T trio with TIM scanner. Four functional runs, one anatomical magnetization-prepared radio-frequency and rapid gradient-echo (MPRAGE) image and one T2 weighted image were acquired for each subject. Prior to performing the functional scans, structural images were collected with 176 slices, dimensions: 224 x 256 x 176 and then resampled with voxel dimensions 1 x 1 x 1 mm, *TR* = 1950 ms. For functional scans, 250 volumes were acquired, with 37 slices per volume. The TR used was 2000 ms, with an interslice time of 54 ms and a TE of 30 ms. Inplane resolution was 64 x 64. Voxel resolution was 3.5 x 3.5 x 3.5 mm, with no slice gap and the flip angle was 90°.

### 2.5. Analysis

The brain imaging data were primarily analyzed using the FSL (Smith et al., 2010) software package. FMRI data processing was carried out using FEAT (FMRI Expert Analysis Tool) Version (version 5.0.1), part of FSL (FMRIBs Software Library, www.fmrib.ox.ac.uk/fsl). Registration to high resolution structural and standard space images was carried out using FLIRT to coregister the participant's structural data to the MNI template space (Jenkinson and Smith, 2001; Jenkinson et al., 2002). The following pre-statistics processing was applied: motion correction using MCFLIRT (Jenkinson et al., 2002), slice-timing correction using Fourier-space time-series phase-shifting, non-brain removal using BET (Smith, 2002), spatial smoothing using a 5.0 mm FWHM Gaussian kernel, grand-mean intensity normalization of the entire 4D dataset by a single multiplicative factor and highpass temporal filtering (Gaussian-weighted least-squares straight line fitting, with sigma = 50.0 s corresponding to a cutoff of a period of 100 s, or 0.01 hz). Time-series statistical analysis was carried out using FILM with local autocorrelation correction (Woolrich et al., 2001). Z (Gaussianised T/F) statistic images were thresholded using clusters determined by *Z*>2.3, corrected for multiple comparisons using random field theory, with a cluster size significance threshold of *p* = 0.05 (Worsley, 2001).

To identify neural correlates of gratitude at the whole brain level, a design matrix was created with four predictor functions in a standard general linear model. The design matrix included predictors for the prime, reflect and probe conditions as well as a parametrically varying predictor for the reflection time period whose height was determined by the level of gratitude reported for each trial. All four runs (corresponding to the phases) were combined using a fixed effects analysis. This parametric regressor was orthogonalized with respect to the main reflection period regressor; thus, the results presented for this regressor represent the variance explained in the blood oxygenation level dependent (BOLD) response by the subjects ratings of gratitude. Ratings were included on a trial-by-trial basis after being mean-corrected for each subject. In a follow-up analyses to visualize the percent BOLD signal chance for each rating in the participants, an ROI was created using the activity found in the MPFC in the whole brain analyses. This ROI was used to interrogate each participant's brain activity for each rating using FSL's Featquery package. The mean percent signal change was extracted for each level from each participant. The mean of all participant's percent signal change was calculated for each rating. In separate analyses, the ratings of need and effort were also used as regressors to examine if and how these ratings explain variance in brain activity. Subject level maps were then fed into a random effects analysis to estimate group level effects.

## 3. Results

Participants rated their gratitude for each gift on a scale of 1–4. The mean of the participants' gratitude ratings was 2.62 (*sd* = 0.334). The participants ratings on the post-experiment questionnaires revealed that participants felt involved in the experiment (*m* = 5.08;*sd* = 1.16), felt that their feelings were similar to if they were in the same situation (*m* = 3.65;*sd* = 1.3) and that they had an increase in their empathy and understanding for the Holocaust (*m* = 4.91;*sd* = 1.33). The participants reported that putting themselves in the situations of the experiment was not very difficult (*m* = 3.04;*sd* = 1.12). See Table 1 for summary. The responses to the open-ended questions indicated that participants did not find any single stimulus to be confusing, that participants did not figure out the experiment on their own, that few participants were trying to figure out the purpose of the study and that no participants had significant prior experience with the Holocaust or with the Shoah Foundation Institute.

**Table 1:** **Responses to post-scan questionnaire**.

**Question**						**95% CI**	
	***t***	***df***	***P***	**Mean**	***sd***	**Lower**	**Upper**
How involved were you?	16.83	22	< 0.001	5.08	1.16	4.58	5.59
How similar were your feelings?	9.78	22	< 0.001	3.65	1.3	3.08	4.21
How much did the experiment increase your empathy for the Holocaust?	16.69	22	< 0.001	4.91	1.33	4.42	5.39
How difficult was it to put yourself in the situations?	−10.97	22	< 0.001	−3.04	1.12	−3.62	−2.47

*The first three questions are compared to the lowest value in the likert scale and the fourth is to the highest value in the scale, since a higher score would mean a greater challenge immersing in the experiment*.

**Figure 4:**
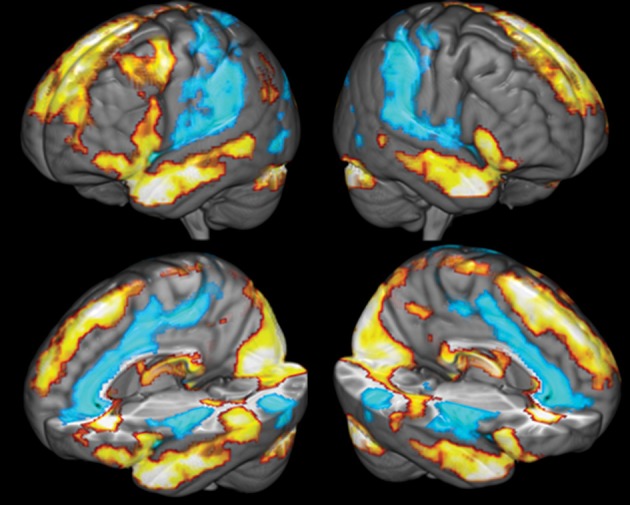
**Comparison of brain activity during the reflection period to baseline**. Yellow colors covering the temporal lobes and superior frontal cortex indicate areas positively associated with the reflection predictor function, blue areas covering the ACC, the insula and secondary somatosensory cortices are negatively correlated with the reflection predictor.

Brain activity was first measured by comparing BOLD activity during the reflection period to baseline to assess participants' general response to the stimuli. The regions positively active during the reflection period, compared to baseline, included the right occipital cortex, the left superior frontal gyrus, the left and right caudate, the left and right temporal pole, the thalamus, the left superior temporal sulcus and the left middle frontal gyrus. Regions that were deactivated included the left and right posterior insula, the right superior temporal gyrus, the perigenual ACC, the right PCC and the left and right middle temporal gyrus (see Figure 4 and Table 2).

**Table 2:** **Brain region peak voxel activity for reflection period compared to baseline**.

**Brain Region**	**Voxels**	***p***	***z*-max**	***z*-max**	***z*-max**	***z*-max**
				***x* (mm)**	***y* (mm)**	***z* (mm)**
**POSITIVE CORRELATION**						
Occipital Cortex	5039	1.69E-33	5.35	16	−102	12
L & R SFG	1390	2.66E-14	5.47	−4	12	70
L Striatum	1026	1.03E-11	5.3	−20	26	16
R Striatum	879	1.39E-10	5.82	18	8	22
L Temporal Pole	809	5.04E-10	5.64	−52	4	−26
R Temporal Pole	405	2.21E-06	5.29	50	12	−32
L & R Thalamus	380	3.93E-06	5.68	0	−28	8
L STS	229	0.0002	4.4	−50	−32	−8
L Posterior MFG	213	0.000317	4.52	−44	6	46
**NEGATIVE CORRELATION**						
Left Insula	1767	9.46E-17	5.15	−40	−6	−12
Right STG	1361	4.19E-14	5.17	64	−26	12
Right Insula	769	1.07E-09	5.48	42	−12	−4
ACC	403	2.26E-06	5.78	0	34	2
Right PMC	303	2.69E-05	4.71	12	−30	46
Right MTS	232	0.000184	4.95	50	−62	6
Left MTS	217	0.000282	4.45	−44	−64	2

*Abbreviations: SFG, superior frontal gyrus; STS, superior temporal sulcus; MFG, middle frontal gyrus; ACC, Anterior Cingulate Cortex; PMC, posteromedial cortex; MTS, middle temporal sulcus. Brain regions, i.e., sulci and gyri, were identified using an neuroanatomy atlas locating the structures at specified MNI coordinates (Damasio, 1995)*.

**Figure 5:**
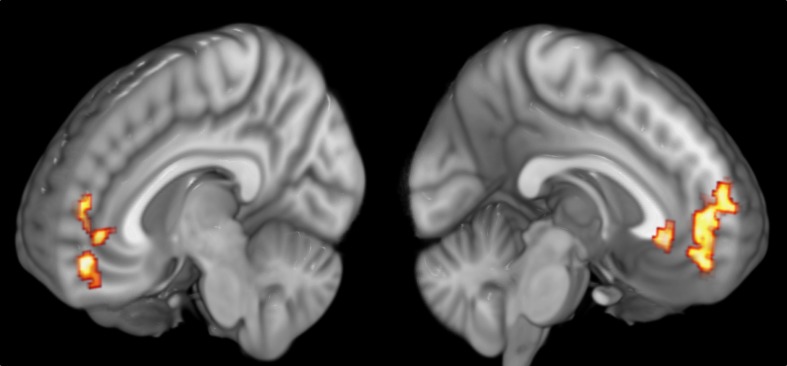
**Medial Prefrontal activity correlating with participants' gratitude ratings**.

**Figure 6:**
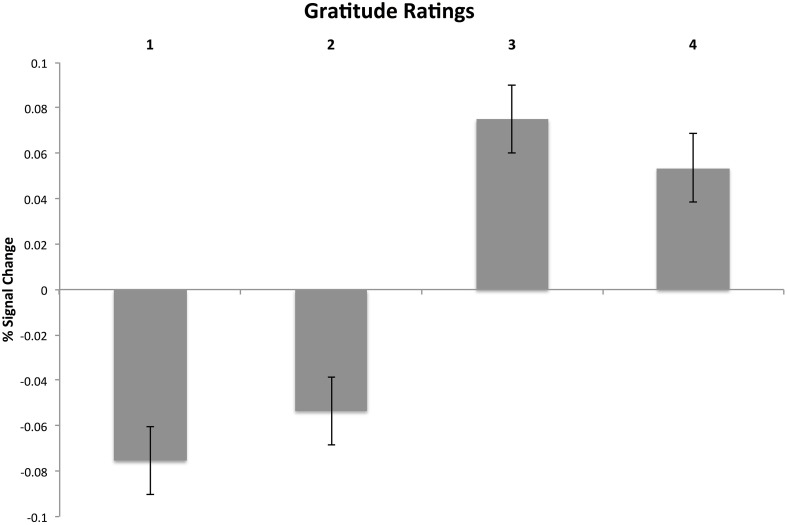
**Visualization of mean-corrected percent signal change for each subject's gratitude ratings during the reflection period**. The signal was extracted from a functionally defined ROI mask of MPFC activity derived from the whole brain GLM analysis of gratitude ratings.

The results showed, at the whole-brain level, that gratitude ratings explained variance in brain activity in a cluster covering multiple regions of the mPFC of both hemispheres (see Figure 5). The cluster included the frontal pole and the peri-genual ACC (*k* = 816;*Z* = 3.48;*x* = −12, *y* = 40, *z* = 4;*p* = 0.009). The local maxima within the cluster included the left perigenual ACC, the right ACC, the left subgenual cingulate cortex, the left and right orbitofrontal cortex and the dorsal mPFC (see Table 3 for summary). To visualize the pattern of results across different gratitude ratings, mean percent signal change for each rating was calculated in each participant using an ROI created by the aforementioned mPFC activity. Percent signal change was calculated using FSL's Featquery tool, which estimates this value by scaling the parameter estimates from the GLM analysis according to the mean signal within the ROI and the peak-to-peak height of the model. Ratings 1 and 2 were marked by an average decrease in activity in the region, and the ratings 3 and 4 were associated with a positive percent signal change (see Figure 6).

**Table 3:** **MNI coordinates of maximum voxel values**.

**Gratitude rating correlates**						
**Cluster Index**	**Voxels**	***p***	***z*****-max**	***z*****-max**	***z*****-max**	***z*****-max**
				**x (mm)**	**y (mm)**	**z (mm)**
MPFC Cluster	816	0.009	3.48	−12	40	4
**LOCAL MAXIMA WITHIN MPFC CLUSTER**						
Left Perigenual ACC			348	12	40	4
Right ACC			3.24	2	54	−8
Left Subgenual ACC			3.11	−2	32	−2
Right OFC			3.11	6	52	−8
Left OFC			3.08	−6	48	4
Dorsal MPFC			3.08	0	56	12

*The top line of data entered denotes the center for the primary cluster found to be active, the lower cells describe the local maxima within the main cluster of activity, revealing activity across sub regions of the MPFC. Abbreviations: MPFC, medial prefrontal cortex; ACC, anterior cingulate cortex; OFC, orbitofrontal cortex*.

Participants also rated each gift according to the level of felt-need and perceived effort. Need significantly correlated with gratitude [*r*_(21)_ = 0.799, *p* < 0.001] and with effort [*r*_(21)_ = 0.342, *p* < 0.001] and effort correlated with gratitude [*r*_(21)_ = 0.508, *p* < 0.001]. These correlations confirm findings from previous studies on the determinants of gratitude (Tesser et al., 1968). Need and effort ratings were independently examined to determine the correlation of each rating with brain activity during the reflection period. Need and effort ratings did not significantly explain variance in brain activity in any region.

## 4. Discussion

This investigation sought to identify neural correlates of gratitude. We hypothesized that ratings of gratitude would correlate with BOLD signal magnitude in brain regions involved in moral cognition (MPFC, ACC), reward (vMPFC), and theory of mind (dorsal MPFC), and basic emotion (insula). In support of the hypotheses, ratings of gratitude correlated with activity in a region of the MPFC that encompassed the peri-genual ACC and the ventral and dorsal MPFC. Activity in these regions has been linked to reward and moral cognitive processes, such as reward from the relief of removing a stressor (Leknes et al., 2013), subjective value judgments (Kringelbach, 2005; D'Argembeau, 2013), fairness and economic decision-making (Tabibnia and Lieberman, 2007; Weber et al., 2009) and processes of self-reference (Denny et al., 2012; Araujo et al., 2013). Experiencing gratitude may coopt the MPFCs general role in evaluating the subjective value of a stimulus and calculating the mental states of others. This interpretation is consistent with previous investigations, meta-analyses and review articles implicating the MPFC in rewarding social interactions, empathic behavior, and theory of mind (Harris et al., 2007; van den Bos et al., 2007; Bzdok et al., 2012; Rameson et al., 2012). This being one of the first such studies of the neural bases of gratitude, interpreting the results presents a challenge. We consider our findings then, in terms of the general role of the MPFC in the domains of moral and social cognition, perspective taking, reward, and basic emotion, discussed in turn below.

Gratitude is often conceived of as a moral emotion (McCullough et al., 2001). Thus, the experience of gratitude should recruit brain regions associated with moral cognition. The maps elicited by Bzdok and colleagues in their meta-analysis showed that morality (studies involving judgments made about the appropriateness of people's actions, as in moral dilemmas) is consistently associated with activity in areas that overlap with those found in our data (2012). They also showed via conjunction analysis that morality, theory of mind and empathy elicited activity in the dorsomedial prefrontal cortex, similar to the regions active in our study. More specifically, their contrast of morality with empathy yielded brain activity in regions related to morality overlapping with our data, more so than the regions associated with empathy. In a related study of receiving help from others, Decety and Porges found that imagining being helped by another person elicited activity in the ACC, dorsomedial and ventromedial PFC and supplementary motor area (2011). There is a large degree of similarity between our study and Decety and Porges (2011), providing support to the notion that our stimuli were successful in eliciting brain activity related to the recognition of help from others, although their study did not address whether participants felt grateful.

Gratitude for gifts is also inherently social. The regions that we find to be active, particularly those in the ventral and subgenual regions of the MPFC, are commonly associated with social reward and interpersonal bonding. Van den Bos and colleagues found that the perigenual-ACC portion of the MPFC is active following rewarding social interactions (2007). The MPFC is also known to be active during social support and pain relief associated with viewing a loved one (Eisenberger et al., 2011). Literature reviews and meta-analyses have implicated the MPFC as a hub for processing the reward of social interactions and affective processing (Tabibnia and Lieberman, 2007; Fareri and Delgado, 2014), and pointed to its general role in binding affective stimuli with related perceptual cues (Shenhav et al., 2013).

It has been said that it is the thought behind a gift that drives gratitude (Ames et al., 2004), so it is reasonable that gratitude in the context of gift-giving will rely on brain circuits associated with theory of mind and emotion perception. The dorsal MPFC is associated with both emotion perception and theory of mind (Mitchell and Phillips, 2015). In our data, the area we see active in the dorsal region of the MPFC corresponds with results found in a meta-analysis of theory of mind and strategic games (Schurz et al., 2014). One review posits that activity in the MPFC is related to the mentalizing content of a stimulus and that the MPFC is likely activated by cognitive reasoning due to the needs to infer social agency and theory of mind (Van Overwalle, 2011).

If gift-giving is partly related to understanding others, it stands to reason that some aspect of self-processing must also be involved. The MPFC is critical for self-processes (Araujo et al., 2013). Activity in the MPFC falls on a spatial gradient moving from ventral regions associated with self-related to dorsal regions associated with other-related judgment (Denny et al., 2012). Interestingly, the data from our study show some overlap with both the “self” and the “other” regions found in Denny et al. (2012), which may inform our conception of gratitude as it emerges from understanding others' minds in conjunction with our own needs.

Finally, gratitude as a social emotion is related to general affective processing. Meta-analyses of neural networks involved in affective processing have found data that overlap with the present study, pointing to gratitude as an emotion at the intersection of social processing and other more general affective processes. In a meta-analysis to determine networks involved in emotional processes, it was found that the MPFC, in a region similar to ours, functioned at the intersection of core affect and cognitive context, and was connected to the core limbic group (Kober et al., 2008). Building on this, others suggest that the MPFC is a neural hub, connected to parasympathetic function and is critical for generating “meaning” in a stimulus (Roy et al., 2012).

Given the important role of the MPFC in perspective-taking, we must consider the possibility that the regions active in our data correlate with task-related perspective-taking demands and not with feelings of gratitude *per se*. The stimuli were designed to involve a more or less uniform amount of context and complexity such that the correspondence between how much gratitude the gifts elicited was not inherently scaled to the amount of perspective-taking needed to understand the gift. We cannot exclude the possibility that participants were better able to generate gratitude when they were successful in perspective-taking. But while that may be the case, it should be noted that effort ratings, which may serve as a proxy for perspective-taking, did not correlate with brain activity. In fact, the ratings for how much a gift was needed were better predictors for the ratings of gratitude overall, which helps minimize the potential confound of perspective-taking as a primary component in explaining variance in brain activity during the experiment.

The gifts in our study are aimed, generally, at restoring life-functions. In other words, the gifts are designed to relieve the recipient of a stressor, to some varying degree. Interestingly, insular activity during the reflection period was decreased compared with the resting baseline. If we conceive of each stimulus as capable of relieving some degree of stress, then perhaps the insula's activity is mapping some aspect of this relief, although it is unclear why activity in the insula was not correlated with gratitude ratings. This is commensurate with recent studies showing that insula activity decreases when pain decreases through analgesia or long-term meditation training, respectively (Schmid et al., 2013; Nakata et al., 2014; Meier et al., 2015). More broadly, given the overlap with our results and investigations of pain and empathy (Singer et al., 2004; Jackson et al., 2006; Lamm et al., 2007), the relationship between gratitude, pain, and empathy may provide important insight into the means by which gratitude is associated with improved health outcomes (Huffman et al., 2014), benefits to relationships (Algoe et al., 2008) and subjective well-being (Emmons, 2008).

One limitation to the study is that the participants did not receive gifts themselves, and instead were asked to imagine the experience. Nevertheless, we believe that participants in our study felt real gratitude for a number of reasons. Participants were told to use their own reactions to rate the stimuli and to feel based on their own perspective; these responses were the bases for the analyses, thus decreasing the chance that experimenter bias would influence their responses. In addition, participants reported that their feelings during the study were similar to what they would have felt if they were in the same situation, that they felt involved in the experiment, that the experiment was not difficult, and even that the experience increased their empathy for and understanding of the Holocaust. Given our study design, the results can also be compared to prior results on gratitude in the context of gift-giving (Tesser et al., 1968; Lane and Anderson, 1976; Wood et al., 2010). These studies used brief scenarios in which the participants were asked to feel how much gratitude they would experience in a given situation. Our paradigm relies on a similar approach, strengthened by the reference to powerful historical events. Our design is also similar to related studies of social emotions such as compassion, admiration and empathy, which used rich and realistic narrative-based stimuli to elicit complex social emotions (Immordino-Yang et al., 2009; Decety and Porges, 2011; Fox et al., 2013). Additionally, reading emotional stories to elicit emotional experiences has been shown to elicit strong and realistic emotional responses (Mar et al., 2011).

In the historical setting of the Holocaust, in which receiving even a small gift could mean another day of survival, our results serve as reminders that in the midst of tragedy there can be acts of compassion, sacrifice, and profound human dignity.

## Author contributions

GF designed the study and conducted the research, analyzed the data, and wrote the manuscript. JK contributed to study design, data analysis and manuscript preparation. HD and AD contributed to study design and manuscript preparation.

## Funding

This study was supported by the Oskar Schindler Humanities Foundation, the Shoah Foundation Institute, and a Dissertation Research Award from the Greater Good Science Center's Expanding the Science and Practice of Gratitude Initiative.

### Conflict of interest statement

The authors declare that the research was conducted in the absence of any commercial or financial relationships that could be construed as a potential conflict of interest.
